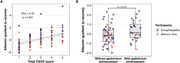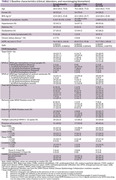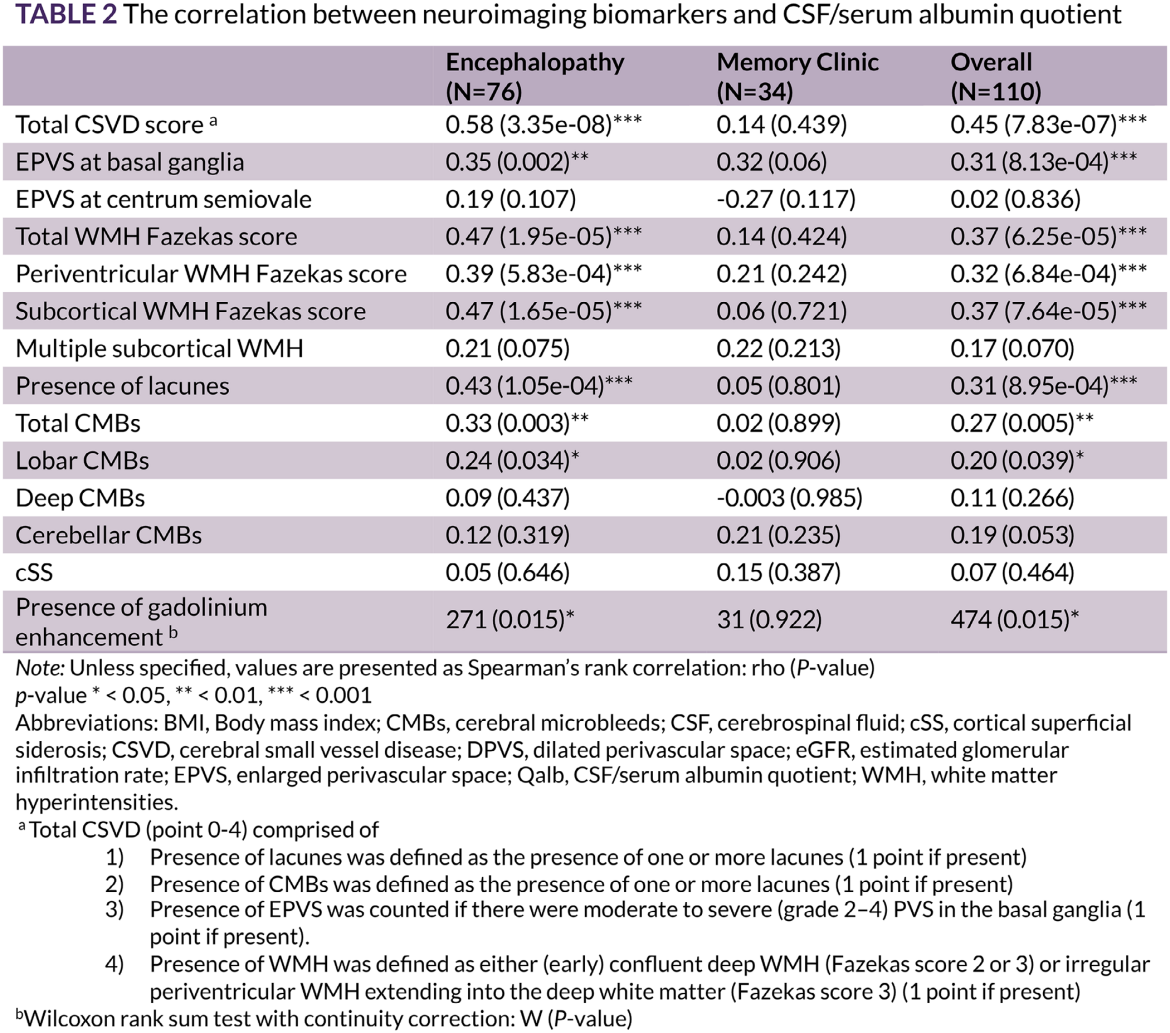# Correlation between cerebral small vessel disease biomarkers and blood‐brain barrier permeability in participants of the encephalopathy and memory clinic cohort

**DOI:** 10.1002/alz.091157

**Published:** 2025-01-09

**Authors:** Thanakit Pongpitakmetha, Poosanu Thanapornsangsuth, Nattanich Pornteparak, Tatchaporn Ongphichetmetha, Juthamas Rianaree, Watayuth Luechaipanit, Thanaporn Haethaisong, Adipa Chongsuksantikul, Sekh Thanprasertsuk, Yuttachai Likitjaroen, Thiravat Hemachudha

**Affiliations:** ^1^ Faculty of Medicine, Chulalongkorn University, Bangkok Thailand; ^2^ Chula Neuroscience Center, King Chulalongkorn Memorial Hospital, Bangkok Thailand; ^3^ Memory Clinic, King Chulalongkorn Memorial Hospital, Bangkok Thailand; ^4^ Thai Red Cross Emerging Infectious Diseases Health Science Centre, King Chulalongkorn Memorial Hospital, Bangkok Thailand; ^5^ Department of Medicine, Banphaeo General Hospital, Samutsakhon Thailand; ^6^ Siriraj Neuroimmunology Center, Faculty of Medicine Siriraj Hospital, Mahidol University, Bangkok Thailand; ^7^ Cognitive, Clinical and Computational Neuroscience (CCCN) Center of Excellence, Chulalongkorn University, Bangkok Thailand; ^8^ Neurocognitive Unit, Division of Neurology, Faculty of Medicine, Chulalongkorn University, Bangkok Thailand

## Abstract

**Background:**

The blood‐brain barrier (BBB) is considered the crucial part of neuroprotection from various neurological insults including infection, inflammation, and neurodegeneration including Alzheimer’s disease (AD). The cerebral small vessel disease (CSVD) pathologies especially cerebral microbleeds (CMBs) and gadolinium enhancement might reflect the disruption of BBB. The correlation between BBB permeability measured by cerebrospinal fluid (CSF)/plasma albumin quotient (Qalb) and CSVD biomarkers is poorly understood. Thus, we aim to further evaluate this correlation in our cohort.

**Methods:**

Pooling participants from preceding studies on individuals with encephalopathy and from patients attending a memory clinic, where both plasma and CSF were recruited. The magnetic resonance imaging (MRI) closest to the Qalb date was independently rated by the trained investigator using STRIVE‐2 criteria (Duering M, et al. Lancet Neurol. 2023) blinded from the clinical information. The composite total CSVD score (Staals J, et al. Neurology. 2014) ranging from 0‐4 was calculated. The presence of gadolinium enhancement was evaluated. The non‐parametric correlations between neuroimaging biomarkers and Qalb were examined.

**Results:**

110 participants were enrolled. Clinical, laboratory, and neuroimaging biomarkers were summarized in Table 1. A notable positive correlation exists between Qalb and total CSVD score in the whole cohort (Spearman rho = 0.45, p‐value = 7.837e‐07) and encephalopathy cohort (Spearman rho = 0.59, p‐value = 3.347e‐08). The Spearman’s correlation between Qalb and each neuroimaging biomarker was shown in Table 2. Wilcoxon rank sum test with continuity correction between Qalb and presence of gadolinium enhancement was significantly difference (W = 474, p‐value = 0.015). The visualized correlation plots were shown in Figure 1.

**Conclusion:**

The total CSVD and Qalb showed a strong correlation in determining the BBB permeability only in encephalopathy population. The CSVD neuroimaging biomarkers and gadolinium enhancement, which reflect the BBB damage and risk of further neurological deterioration, should carefully be evaluated and considered in clinical practice. The strategies to slow the progression of CSVD including the optimized blood pressure control, avoiding the potential neurotoxic agents, etc. should be raised in this situation, resulting in slowing neuropathological progression. Further confirmatory validation studies, especially memory clinic settings, should be done.